# Need for Real-World Evaluation of Malaria Vaccines and Reliable Vaccination Records in Africa

**DOI:** 10.4269/ajtmh.25-0501

**Published:** 2025-12-30

**Authors:** Yura K. Ko, Tobias Alfvén, Gordon O. Okomo, Wataru Kagaya, Kayoko Shioda, Akira Kaneko, Jesse Gitaka

**Affiliations:** ^1^ Department of Microbiology, Tumor and Cell Biology, Karolinska Institutet, Stockholm, Sweden;; ^2^ Department of Vector Ecology and Environment, Institute of Tropical Medicine, Nagasaki University, Nagasaki, Japan;; ^3^ Department of Global Public Health, Karolinska Institutet, Stockholm, Sweden;; ^4^ Sachs’ Children and Youth Hospital, Stockholm, Sweden;; ^5^ Department of Health, Homa Bay County, Homa Bay, Kenya;; ^6^ Department of Ecoepidemiology, Institute of Tropical Medicine, Nagasaki University, Nagasaki, Japan;; ^7^ Department of Global Health, School of Public Health, Boston University, Boston, Massachusetts;; ^8^ Center on Emerging Infectious Diseases, Boston University, Boston, Massachusetts;; ^9^ Osaka International Research Center for Infectious Diseases/Graduate School of Medicine, Osaka Metropolitan University, Osaka, Japan;; ^10^ Center for Research in Infectious Diseases, Directorate of Research and Innovation, Mount Kenya University, Thika, Kenya;; ^11^ Center for Malaria Elimination, Mount Kenya University, Thika, Kenya

## Abstract

Evaluating the real-world effectiveness of the newly approved malaria vaccines, RTS,S/AS01 and R21/Matrix-M, is critical for informing vaccine policy, especially in areas not represented in the original clinical trials. Observational study designs such as cohort studies using the target trial emulation framework or the test–negative design offer promising approaches for estimating vaccine effectiveness in the real world. However, both designs require accurate, individual–level vaccination data, which remains a major challenge in many African countries. Strengthening electronic immunization registries, alongside continued efforts to improve the quality and completeness of paper-based immunization records, is essential in African countries, not only for the evaluation of current vaccines such as RTS,S/AS01 and R21/Matrix-M, but also in preparation for future malaria vaccines, to support robust vaccine monitoring and decision-making.

## INTRODUCTION

Malaria is still a major health problem, particularly in Africa, where 95% of global malaria mortality occurs. According to the World Malaria Report 2024, the estimated case incidence in the region in 2023 was 226.8 per 1,000 population.^1^ Although the morbidity and mortality of malaria continued to decline from the 2000s to 2015, driven by substantial investments and interventions, including long-lasting insecticide-treated nets (LLINs), indoor residual spraying (IRS), rapid diagnostic tests (RDTs), and artemisinin-based combination therapy, progress has stalled since 2015. Moreover, especially after 2020, it has been reported that malaria incidence and mortality have increased in many African countries.

Currently, two malaria vaccines, RTS,S/AS01 and R21/Matrix-M, are recommended by the World Health Organization, with several other candidates undergoing phase 1 or 2 clinical trials.^2^ In 2024, 14 African countries introduced either the RTS,S/AS01 or R21/Matrix-M vaccines, in addition to Ghana, Kenya, and Malawi, which initially piloted the RTS,S/AS01 implementation in 2019.^2^ The recommended regimens and schedules are similar for both vaccines. The first dose is administered at around the age of 5 months, followed by the second and third doses at least one month apart. The fourth dose is given 6–18 months after the third to extend protection.^3^

### Need for real-world evaluation of malaria vaccines.

Evaluating the real-world vaccine effectiveness after implementing large-scale immunization programs is essential, particularly in regions not included in the original clinical trials. Variations in vaccination schedules, cold–chain logistics, health system infrastructure, transmission level, and implementation of other interventions across African countries may significantly affect vaccine effectiveness in practice. In particular, the World Health Organization underscores postlicensure studies on vaccine effectiveness of the R21/Matrix-M malaria vaccine in high perennial transmission settings as a key research priority.^3^ Furthermore, no head-to-head comparison between RTS,S/AS01 and R21/Matrix-M has been conducted. In phase 3 clinical trials conducted at 11 sites across 7 African countries, the RTS,S/AS01 vaccine showed an efficacy of 55% (95% CI, 51–59) against multiple episodes of clinical malaria during the first year of follow-up among children aged 5–17 months.^4^ Meanwhile, the R21/Matrix-M vaccines demonstrated efficacies of 75% (95% CI 71–78) at sites with seasonal transmission and 67% (95% CI, 59–73) at sites with perennial transmission, where age-based immunization was implemented among children aged 5–36 months in its phase 3 trial.^5^ Although R21/Matrix-M appears more efficacious, Macià et al.^6^ recently highlighted the challenges of comparing the two vaccines due to differences in the trials’ design, the interaction between waning vaccine protection and seasonal transmission patterns, and other contextual factors. In 2024, Ghana began deploying both vaccines,^2^ whereas Kenya plans to introduce R21/Matrix-M in addition to RTS,S/AS01, which has been in use since 2019. These countries will offer a unique opportunity to directly compare the two vaccines under real-world conditions. Moreover, in high-transmission settings, previous studies have reported a rebound in malaria incidence among vaccinated older children, likely due to reduced opportunities to develop naturally acquired immunity.^7^ Although the extent of malaria rebound has not been shown to outweigh the benefits of malaria interventions,^3^ long-term monitoring of this potential rebound remains important.

### Malaria vaccine evaluation in observational studies.

Given that randomized controlled trials are practically challenging, observational study designs can be a useful and necessary alternative to estimate vaccine effectiveness. After the emergence of coronavirus disease 2019 (COVID-19), many countries incorporated modern causal inference frameworks, including target trial emulation (TTE), to evaluate the real-world effectiveness of vaccines using observational data.^8^ When carefully designed, cohort designs using observational data can emulate a hypothetical target trial and estimate causal effects of vaccination.^9^ Within the TTE framework, clearly specifying the time zero for both treatment and control groups minimizes selection bias and immortal time bias, which are common challenges in estimating the causal effects of interventions using observational data.^9^ Consequently, TTE has gained attention as a practical design for evaluating interventions.^8^ Furthermore, longitudinal cohorts allow the detection of rare or long-term outcomes, which are difficult to capture in randomized controlled trials.^10^ In particular, they enable the estimation of vaccine effectiveness against death using the TTE framework.^8^ However, global disparity remained in the application of TTE; most TTE-based vaccine evaluations have been conducted in the United States and Europe,^8^ primarily because this approach requires high-quality surveillance and vaccine registry databases that can be linked together at the individual level.^11^ To conduct TTE for malaria vaccines, robust vaccination data linked with infection or clinical outcomes are necessary. Establishing such surveillance systems is particularly challenging in many low- and middle-income countries because of limited resources and population migration.

In settings where such databases are unavailable and establishing a cohort is not feasible, the test–negative design (TND) offers a practical alternative.^12^ Since the 2020 pandemic, TND has also been increasingly used, including in African studies of COVID-19 vaccine effectiveness.^13^ Its main advantage is efficiency, because it avoids the need for longitudinal follow-up. This feature makes it particularly suitable in settings where continuous individual tracking is difficult. TND may also help reduce confounding by limiting the study population to individuals who seek care and are tested for the target disease.^12^ Li et al.^14^ reported that when rich data on demographic characteristics, medical factors, and healthcare utilization were available, vaccine effectiveness estimates for the BNT162b2 COVID-19 vaccine were similar using both a TND and a cohort design with explicit TTE. In malaria-specific contexts, these study designs must additionally account for confounding due to other control measures such as LLIN and IRS, and other preventive behaviors. Furthermore, the potential for misclassification of disease outcomes, resulting from the imperfect sensitivity and specificity of diagnostic tests, needs to be considered, particularly in malaria-endemic settings where RDTs are most commonly used.^15^

### Vaccine records in Africa.

In both study designs, accurate individual–level vaccination records are essential for the implementation. Several approaches are available for tracking vaccination information. In malaria-endemic regions, home-based records remain the most accessible tool.^16^ In particular, maternal and child health handbooks provide essential guidance on malaria prevention, including immunization, along with information on pregnancy care, childbirth, early childhood, nutrition, and other key health practices.^16^ However, they are primarily designed for preschool-aged children and are frequently lost as children grow older. In our 2024 community cross-sectional survey conducted in Homa Bay County, Kenya,^17^ 71% (154/554) of children under the age of 5 years possessed the booklet compared with 43% (278/645) of those aged 5–10 years (Y. K. Ko, unpublished data). This presents a particular challenge for evaluating the aforementioned rebound effects of vaccination in older children. Many African countries have traditionally relied on paper-based immunization registries, which are not easy to access or analyze and are also prone to loss or damage.^18^ Increasingly, governments are transitioning to electronic immunization registries (EIRs), which have the potential to convert immunization records into actionable data for informed decision-making. Countries such as Tanzania and Zambia have already adopted EIRs.^19^ However, several challenges remain, including limited digital infrastructure, high data costs, network instability, data loss, and concerns regarding data quality and interoperability, defined as the ability of systems to exchange and use information effectively, all of which are major barriers to realizing the full potential of EIRs in low-resource settings.^20^ Given these constraints, EIRs should be implemented in a context–appropriate and phased manner, alongside continued efforts to strengthen the quality and completeness of paper-based immunization data. Ensuring accurate individual–level vaccination records also offers the additional advantage of supporting point-of-service tracking, enabling parents and healthcare workers to monitor children’s vaccination schedules and ensure timely receipt of all required doses. This feature is particularly valuable for malaria vaccines such as RTS,S/AS01 and R21/Matrix-M, which both require a fourth dose administered 12–18 months after the third. Coverage of this booster dose often declines substantially, making targeted tracking and follow-up essential for maximizing vaccine effectiveness.

## CONCLUSION

Ensuring the availability of individual-level vaccination records in African countries is essential, not only for evaluating current vaccines such as RTS,S/AS01 and R21/Matrix-M, but also to prepare for future malaria vaccines and to support robust vaccine monitoring and decision-making. These efforts align with the initiatives of Gavi and national governments to strengthen digital health infrastructure and improve immunization delivery for other vaccine-preventable diseases.
